# Recent advances in managing chronic HCV infection: focus on therapy in patients with severe liver disease

**DOI:** 10.12688/f1000research.7399.1

**Published:** 2016-03-17

**Authors:** Raoel Maan, Adriaan J. van der Meer

**Affiliations:** 1 Department of Gastroenterology and Hepatology, Erasmus MC University Medical Center Rotterdam, Rotterdam, 3015, Netherlands

**Keywords:** HCV, Hepatitis C Virus, hepatitis treatment, Liver disease, Chronic hepatitis C virus, interferon

## Abstract

Chronic hepatitis C virus (HCV) infection still represents a major public health problem, as it is thought to be responsible for more than 350,000 deaths around the globe on a yearly basis. Fortunately, successful eradication of the virus has been associated with improved clinical outcome and reduced mortality rates. In the past few years, treatment has improved considerably by the implementation of direct-acting antivirals (DAAs). From 2014 onwards, sofosbuvir, simeprevir, daclatasvir, ledipasvir, paritaprevir, ombitasvir, and dasabuvir have been approved by the US Food and Drug Administration (FDA) and European Medicines Agency (EMA). Regimens with various combinations of these new drugs, without the use of interferon (IFN), proved to be very effective and well tolerated, even among patients with advanced liver disease. Moreover, treatment duration could be shortened to 12 weeks in the majority of patients. The high costs of these DAAs, however, limit the availability of IFN-free therapy worldwide. Even in wealthy countries, it is deemed necessary to prioritize DAA treatment in order to limit the immediate impact on the health budget. As patients with advanced liver disease are in most need of HCV clearance, many countries decided to treat those patients first. In the current review, we focus on the currently available IFN-free treatment options for patients with cirrhosis. We discuss the virological efficacy as well as the clinical relevance of these regimens among this specific patient population.

## Natural history

Chronic hepatitis C virus (HCV) infection continues to be a major global public health problem, with recent estimates suggesting that 64–103 million people are infected worldwide ^1^. Chronic infection leads to slowly progressive hepatic fibrosis, which may eventually lead to cirrhosis ^2, 3^. Once cirrhosis is established, patients have an increased risk of developing complications such as ascites, spontaneous bacterial peritonitis, hepatic encephalopathy, variceal bleeding, and hepatocellular carcinoma (HCC). Although the incidence of HCV infection is declining in the West, it has been estimated that the incidence of patients with HCV-induced cirrhosis will not peak until 2030 ^4^. At the moment, chronic HCV infection is already the leading indication for liver transplantation in many Western countries ^5^. Not to be forgotten, however, is that the natural history of chronic HCV infection extends beyond the liver as well. Before the stage of cirrhosis, there may already be extrahepatic manifestations that impair the patient’s health-related quality of life (HRQoL), of which fatigue is most frequently reported (approximately 50% of patients) ^6– 8^. In terms of solid clinical endpoints, patients are at increased risk of diabetes mellitus, renal failure, cardiovascular events, and malignant lymphoma ^9^. The impaired overall survival among those with chronic HCV infection is thus the result of an increase in both liver-related as well as non-liver-related deaths, as was recently highlighted in an unique natural history study from Taiwan which included 19,636 participants who were followed for a mean duration of 16.2 years ^10^.

## Antiviral therapy

Before 2011, treatment for chronic HCV infection depended on the administration of pegylated interferon alpha (PegIFN) and ribavirin (RBV), which was accompanied by the occurrence of many side effects such as flu-like symptoms, depression, and cytopenias. These side effects were bothersome, especially because the IFN-based regimens had a limited chance of attaining a sustained virological response (SVR [HCV RNA negativity in the circulation 12–24 weeks after cessation of antiviral therapy]). If physicians were not already reluctant to treat out of fear for severe adverse events in the specific population of patients with advanced liver disease, PegIFN and RBV were often unsuccessful. In patients with compensated cirrhosis, SVR rates ranged from 10 to 44% for HCV genotypes 1 and 4 and 33 to 72% for HCV genotypes 2 and 3. When patients were known to have decompensated cirrhosis, SVR rates even dropped to 0–16% for HCV genotypes 1 and 4 and 44–57% for HCV genotypes 2 and 3 ^11^. However, because of safety issues, IFN therapy, in case of unstable liver disease, was mostly restricted to specialized centers. As a result, many patients were unable to attain an SVR, which is considered to be the marker for viral clearance based on its long-term durability ^12^.

The successful development of protease inhibitors for the treatment of the human immunodeficiency virus initiated the development of the first direct-acting antivirals (DAAs) for the treatment of HCV infection. In 2011, the protease inhibitors telaprevir and boceprevir were the first DAAs to be introduced. When added to PegIFN and RBV, the duration of therapy could be halved to 24–28 weeks in about 50% of patients, while SVR rates improved substantially in both treatment-naïve and treatment-experienced patients with HCV genotype 1 ^13– 17^. Unfortunately, among those with cirrhosis, the treatment duration could not be easily reduced and the improvement in the rate of SVR was only limited with an increase to approximately 50%. The downsides include the following: these first two DAAs were not very effective against HCV genotypes other than genotype 1; treatment became more complex with various dosing schedules, durations, and stopping rules; the pill burden was large; the rates of resistance-associated variants (RAVs) were high; and there were many potential drug-drug interactions. Moreover, the first real-world data raised important safety issues, especially among patients with compensated cirrhosis, and PegIFN remained a necessity ^18^. The development of antiviral therapy has moved at an incredible pace during the 3 years following the first proof-of-concept that chronic HCV infection could be eradicated without PegIFN ^19^. At the moment, IFN-free regimens, in which multiple classes of DAAs are combined, revolutionize the treatment of chronic HCV infection. Short and well-tolerated regimens have reported SVR rates of around 95%, even among patients with cirrhosis ^20– 24^. Unfortunately, the high costs of the DAAs currently make these drugs unavailable for the majority of patients worldwide. Also, in wealthy countries it is deemed necessary to prioritize DAA treatment in order to limit the immediate impact on the health budget, even though modeling data indicated that the IFN-free regimens are cost effective in the long term. As a consequence, physicians are often limited to treat only those patients with advanced liver disease, the specific population on which we will focus in the current review.

## Life cycle of the hepatitis C virus

**Figure 1.**
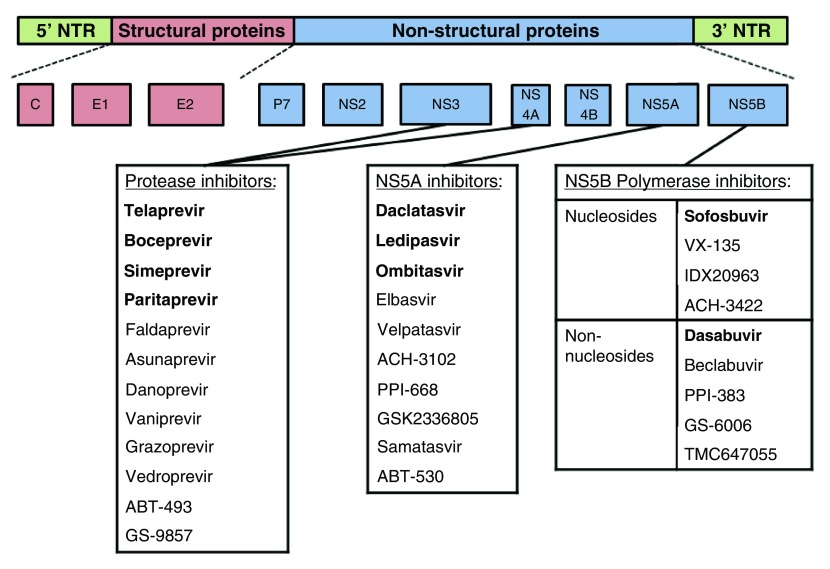
The hepatitis C virus (HCV) genome. The hepatitis C virus (HCV) genome encoding three structural proteins and seven non-structural proteins. The direct-acting antivirals are listed below the proteins and include the NS3/4A (or protease) inhibitors, the NS5A inhibitors, and the NS5B polymerase inhibitors (both nucleosides and non-nucleosides). The direct-acting antivirals approved by the US Food and Drug Administration and the European Medicines Agency are highlighted in bold.

**Figure 2.**
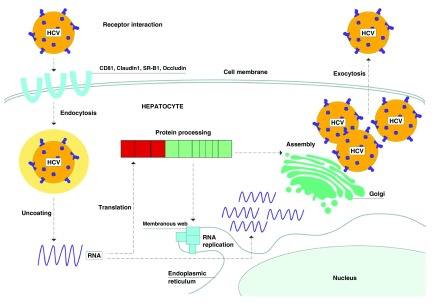
Life cycle of the hepatitis C virus. Adapted from Feeney *et al.* ^26^. Schematic overview of the life cycle of the hepatitis C virus (HCV). In order to enter the hepatocyte, HCV interacts with co-receptors, resulting in its endocytosis. Then the virus fuses with the endosome and uncoats its RNA. Host ribosomes translate the RNA into a polyprotein, which is cleaved by host and virally encoded proteases into the three structural and seven non-structural proteins. The non-structural proteins form a complex on a “membranous web” that replicates HCV RNA. The Golgi assembles the HCV RNA with viral structural proteins, leading to the formation of infectious viral particles, which are exocytosed from the cell. © 2015 BMJ Publishing Group Ltd. All rights reserved.

Hepatitis C virus is a small enveloped virus of approximately 55–65 nanometers in size and is a member of the genus Hepacivirus, belonging to the *Flaviviridae* family. It contains a single-stranded RNA genome of positive polarity. This genome is approximately 9600 nucleotides in length and consists of a highly conserved 59 untranslated region, followed by a single open reading frame that encodes a polyprotein of 3010 to 3033 amino acids. Cellular and viral proteases cleave this large protein into ten smaller viral gene products: three structural proteins (core, E1, and E2); an ion channel (p7); and six nonstructural proteins (NS2, NS3A, NS4A, NS4B, NS5A, and NS5B) ( Figure 1). Structural proteins are required for assembly and are used for the determination of the seven main HCV genotypes (and subgenotypes) ^5^. The p7 and NS2 protease are required for the release of infectious particles. The other nonstructural proteins (NS3A, NS4A, NS4B, NS5A, and NS5B) are closely involved in HCV replication ^25^. NS3 and its cofactor NS4A form a stable heterodimeric complex, which cleaves the HCV polyprotein at four sites. NS4B is the presumed central organizer of the HCV replicase complex and a main inducer of intracellular membrane rearrangements. The NS5A protein is essential for RNA replication and assembly of infectious virus particles. The RNA-dependent NS5B protein is the RNA polymerase catalyzing the amplification of the viral RNA genome ^25, 26^. Figure 2 shows the entry of HCV into the hepatocytes, as well as its life cycle and replication process ^26^. In addition, several host factors have been involved in the HCV life cycle, which may represent new targets for antiviral treatment. These include epidermal growth factor receptor (EGFR) and ephrin receptor A2 (EphA2), which are two receptor tyrosine kinases that have recently been identified as HCV entry factors ^27^. Another host factor, microRNA-122 (miR-122), is a hepatocyte-abundant microRNA which binds to the 5’ untranslated region of the HCV genome. Hereby, it is thought to promote HCV RNA stability and accumulation and to protect the HCV genome from the innate immune response ^28^. Cyclophilin A (CypA) is a protein that is involved in the replication of HCV by binding to the NS5A protein of all HCV genotypes ^29^. Lastly, apolipoprotein E (apoE) is a component of lipoviral particles, which is involved in the HCV infection of hepatocytes ^30^.

## Mechanism of action of antiviral drugs

Although still not fully elucidated, IFN is thought to induce a large number of genes (called IFN-stimulated genes) with antiviral properties, leading to a multi-faceted attack on the virus. In addition, it also has some direct antiviral actions as well as important interactions with the adaptive and innate immune responses ^31^. RBV is a guanosine analogue with activity against several RNA and DNA viruses. Different hypotheses regarding its mechanism of action have been proposed, of which the theory of lethal mutagenesis seems most reasonable ^31^. Protease inhibitors target the NS3/4A serine protease and thereby inhibit the cleavage of this protein and thus HCV replication ^26^. Current approved “- **p**revirs” include telaprevir, boceprevir, simeprevir, and paritaprevir ( Figure 1). The NS5A inhibitors, also known as “- **a**svirs”, target another nonstructural protein and block the replication of HCV RNA at the stage of membranous web biogenesis ^32^. So far, daclatasvir, ledipasvir, and ombitasvir have been approved ( Figure 1). Both NS3/4A protease inhibitors and NS5A inhibitors have very potent antiviral activity but exhibit a low barrier to viral resistance. The NS5B inhibitors, or “- **b**uvirs”, can be divided into two main classes: nucleos(t)ide inhibitors and non-nucleotide inhibitors ( Figure 1). By binding to the active site of the NS5B RNA-dependent RNA polymerase, nucleos(t)ide inhibitors (e.g., sofosbuvir) cause premature chain termination. The non-nucleotide inhibitors (e.g., dasabuvir) bind outside the active site, causing a conformational change, and thereby decrease the polymerase activity of the enzyme ^26^.

## Current treatment regimens

There are extensive data from phase III or IV studies on the efficacy of IFN-free regimens, but patients with compensated and decompensated cirrhosis were often underrepresented. Currently, more data are emerging from real-world studies on the efficacy of these regimens, which included patients with the most severe cirrhosis. Table 1– Table 4 show the data from phase III or IV studies available on the efficacy of IFN-free antiviral therapy. Below, we will discuss the treatment regimens in a more conceptual way for the treatment of patients with compensated cirrhosis.

**Table 1.** Rates of SVR for patients with compensated cirrhosis HCV genotype 1 (phase III/IV studies).

Regimen	Duration	Patients	SVR rate	Study (reference)
LDV – SOF ± RBV	12–24 weeks	Naïve	97–100%	ION-1 ^21^
LDV – SOF ± RBV	12–24 weeks	TE	82–100%	ION-2 ^20^
LDV – SOF LDV – SOF - RBV	24 weeks 12 weeks	TE TE	97% 96%	SIRIUS ^84^
SIM – SOF	12 weeks	Naïve TE	88% 79%	OPTIMIST-2 ^22^
SIM – SOF ± RBV	12 weeks	Naïve TE	75% 73–88%	Trio Network ^85^
SIM – SOF ± RBV	12 weeks	Naïve TE	87–93% 80–82%	HCV-Target ^86^
OMB – PAR/r – DSV + RBV	12–24 weeks	Naïve TE	94–95% 90–97%	TURQUOSE-II ^23^
OMB – PAR/r – DSV	12 weeks	TE	100%	TURQUOSE-III ^24^
GPV – ELB	12 weeks	Naïve	97%	C-EDGE ^87^
SIM – SOF	12 weeks	Naïve TE	95% 92%	Pearlman *et al.* ^88^
SIM – SOF	12 weeks	Naïve TE	83%	Aqel *et al.* ^89^
SIM – SOF ± RBV	12 weeks	Naïve TE	91–94% 88–95%	Saxena *et al.* ^46^
SIM – SOF	12 weeks	Naïve and TE	87%	Shiffman *et al.* ^90^
DCV – ASV – BCV ± RBV	12 weeks	Naïve and TE	87–98%	Unity-2 ^91^
VPV – SOF	12 weeks	Naïve and TE	99%	ASTRAL-1 ^45^

a. Abbreviations: ASV, asunaprevir; BCV; beclabuvir; DCV, daclatasvir; DSV, dasabuvir; ELB, elbasvir; GPV, grazoprevir; HCV, hepatitis C virus; LDV, ledipasvir; OMB, ombitasvir; PAR/r, paritaprevir/ritonavir; RBV, ribavirin; SIM, simeprevir; SOF, sofosbuvir; SVR, sustained virological response; TE, treatment-experienced; VPV, velpatasvir

**Table 2.** Rates of SVR for patients with compensated cirrhosis HCV genotype 2–4 (phase III/IV studies).

HCV Genotype	Regimen	Duration	Patients	SVR rate	Study (reference)
HCV genotype 3	DCV – SOF	12 weeks	Naïve and TE	58–97%	ALLY-3 ^43^
HCV genotype 2 HCV genotype 3	SOF + RBV	12 weeks 12 weeks	Naïve	91% 34%	FISSION ^40^
HCV genotype 2 HCV genotype 3	SOF + RBV	12 weeks 12 weeks	Naïve	94% 21%	POSITRON ^39^
HCV genotype 3 HCV genotype 2 HCV genotype 3	SOF + RBV	24 weeks 12 weeks 24 weeks	Naïve TE TE	92% 78% 62%	VALENCE ^92^
HCV genotype 2 HCV genotype 3 HCV genotype 3	SOF + RBV	16 weeks 24 weeks 16 weeks 24 weeks 16 weeks 24 weeks	TE Naïve TE	87% 100% 57% 82% 47% 76%	BOSON STUDY ^44^
HCV genotype 2	SOF + RBV	12 weeks	Naïve TE	67% 76%	Trio Network ^85^
HCV genotype 2 HCV genotype 3	SOF + RBV SOF + RBV	12 weeks 16 weeks 12 weeks 16 weeks	TE TE	60% 78% 19% 61%	FUSION ^39^
HCV genotype non-1	SOF – VPV	12 weeks	Naïve and TE	100%	ASTRAL-1 ^45^
HCV genotype 2	SOF – VPV	12 weeks	Naïve and TE	100%	ASTRAL-2 ^93^
HCV genotype 3	SOF – VPV	12 weeks	Naïve and TE	91%	ASTRAL-3 ^93^
HCV genotype 3	DCV – SOF + RBV	12 weeks 16 weeks	Naïve and TE	83% 89%	ALLY-3+ ^94^

a. Abbreviations: DCV, daclatasvir; HCV, hepatitis C virus; RBV, ribavirin; SOF, sofosbuvir; SVR, sustained virological response; TE, treatment-experienced; VPV, velpatasvir

**Table 3.** Rates of SVR for patients with decompensated cirrhosis HCV genotype 1 (phase III/IV studies).

Regimen	Duration	Patients	SVR rate	Study (reference)
LDV – SOF ± RBV	12 – 24 weeks	Naïve and TE	86–89%	SOLAR-1 ^82^
LDV – SOF ± RBV	12 – 24 weeks	Naïve and TE	84–92%	SOLAR-2 ^95^
SOF + RBV SIM – SOF SIM – SOF + RBV	12 weeks 12 weeks 12 weeks	Naïve and TE Naïve and TE Naïve and TE	52% 74% 66%	HCV-Target ^96^
DCV – SOF + RBV	12 weeks	Naïve and TE	56–94% ^*^	ALLY-1 ^97^
LDV – SOF LDV – SOF + RBV DCV – SOF DCV – SOF + RBV	12 weeks 12 weeks 12 weeks 12 weeks	Naïve and TE Naïve and TE Naïve and TE Naïve and TE	81% 86% 60% ^#^ 82%	UK cohort EAP ^81^
SIM – SOF ± RBV	12 weeks	Naïve and TE	69–79%	Saxena *et al.* ^46^
SIM – SOF ± RBV	12 weeks	Naïve and TE	68%	Aqel *et al.* ^89^
SIM – SOF	12 weeks	Naïve and TE	67–77%	Shiffman *et al.* ^90^
SIM – SOF SIM – SOF + RBV	12 weeks 12 weeks	Naïve and TE Naïve and TE	52–65% 44–65% ^#^	Backus *et al.* ^98^
SOF – VPV SOF – VPV + RBV SOF – VPV	12 weeks 12 weeks 24 weeks	Naïve and TE	88% 96% 92%	ASTRAL-4 ^99^

a. Abbreviations: DCV, daclatasvir; HCV, hepatitis C virus; LDV, ledipasvir; RBV, ribavirin; SIM, simeprevir; SOF, sofosbuvir; SVR, sustained virological response; TE, treatment-experienced; VPV, velpatasvir

b. ^*^Also included patients with HCV genotype 2, 3, and 4

c. ^#^Fewer than 10 patients were included in a specific subgroup

**Table 4.** Rates of SVR for patients with decompensated cirrhosis HCV genotype non-1 (phase III/IV studies).

HCV genotype	Regimen	Duration	Patients	SVR rate	Study (reference)
HCV genotype 4	LDV – SOF ± RBV	12 – 24 weeks	Naïve and TE	57–86%	SOLAR-2 ^95^
HCV genotype 2 HCV genotype 3	SOF + RBV	12 weeks	Naïve and TE	81% 39%	HCV-Target ^96^
HCV genotype 2 HCV genotype 3 HCV genotype 4	DCV – SOF + RBV	12 weeks	Naïve and TE	80% ^#^ 83% ^#^ 100% ^#^	ALLY-1 ^97^
HCV genotype 3 Other HCV genotypes than 1 and 3	LDV – SOF ± RBV DCV – SOF ± RBV LDV – SOF + RBV DCV – SOF ± RBV	All 12 weeks	Naïve and TE	43–59% ^#^ 70–71% ^#^ 89% 85–100% ^#^	UK cohort EAP ^81^
HCV genotype 2	SOF + RBV	12 weeks	Naïve and TE	44–71% ^#^	Backus *et al.* ^98^
HCV genotype non-1	SOF – VPV SOF – VPV + RBV SOF – VPV	12 weeks 12 weeks 24 weeks	Naïve and TE	50–100% ^#^ 85–100% ^#^ 50–100% ^#^	ASTRAL-4 ^99^

a. Abbreviations: DCV, daclatasvir; HCV, hepatitis C virus; LDV, ledipasvir; RBV, ribavirin; SIM, simeprevir; SOF, sofosbuvir; SVR, sustained virological response; TE, treatment-experienced

b. *Also included patients with HCV genotype 2, 3, and 4

c. ^#^Fewer than 10 patients were included in a specific subgroup

### HCV genotype 1

In the Western world, HCV genotype 1 is the most prevalent (>50%). The initial development of DAAs was therefore mainly focused on this genotype. Although IFN-free therapy is preferred, the combination of a second-generation NS3/4A protease inhibitor, a NS5A inhibitor, or a NS5B inhibitor with PegIFN and RBV for 12–48 weeks has been assessed ^33– 36^. Although not approved, even the addition of a NS3/4A protease inhibitor and a NS5A inhibitor to PegIFN and RBV for 24 weeks could have been an option for the treatment of chronic HCV genotype 1 infection ^37^. For patients who are unable to tolerate PegIFN, the combination of a NS5B inhibitor and RBV was assessed, but phase III studies were never performed due to the lack of efficacy. When focusing on the regimens that did reach SVR rates of more than 90%, the optimal regimen consists of at least two classes of DAAs, with or without the addition of RBV. The combination of a NS5B inhibitor with a NS5A inhibitor and/or a NS3/4A protease inhibitor is enough to create a high barrier to resistance ^20– 22^. In cirrhotic patients, some regimens show lack of efficacy, which could be improved by the addition of RBV and/or the extension of antiviral therapy to 24 weeks. As the development of novel DAAs is still ongoing, recent data have shown that second-generation regimens, including a NS5A inhibitor and a NS3/4A protease inhibitor, may be equally effective for this genotype ^38^. Thus, inclusion of a NS5B inhibitor may not be a necessity.

Since there is a difference in efficacy among patients with HCV genotype 1a and 1b, different regimens were applied among these patients ^23, 24^. RAVs that are present at baseline or emerge during antiviral therapy may account for this difference between the two subtypes. Therefore, before initiating most regimens, subtyping of the HCV genotype 1 is required. However, lower response rates in patients with HCV genotype 1a seem to be a problem only when a NS3/4A protease inhibitor is incorporated into the treatment regimen ^20, 21, 23^. At the price of additional side effects, this effect may be partly overcome by adding RBV to the treatment regimen.

### HCV genotype 2

Historically, patients with HCV genotype 2 were the easiest to treat, even when patients had cirrhosis. Currently, an IFN-free combination including a NS5B inhibitor and RBV seems sufficient to clear the virus ^39– 41^. When patients are intolerant to RBV, a regimen with a NS5B inhibitor and a NS5A inhibitor could be an attractive option, as both have antiviral activity against this genotype. This combination, however, has not been extensively investigated in clinical trials ^42^.

### HCV genotype 3

Patients with HCV genotypes 2 and 3 were found to be relatively IFN sensitive and required a shorter duration of therapy with lower doses of RBV to achieve higher rates of SVR as compared to patients with HCV genotypes 1 and 4. Even with PegIFN and RBV, however, HCV genotype 3 was more difficult to cure than genotype 2, particularly in patients with established cirrhosis. In the current IFN-free era, HCV genotype 3 has actually replaced HCV genotype 1 as the most challenging genotype. In contrast to its effect in non-cirrhotic patients with HCV genotype 3, the combination of a NS5B inhibitor and RBV in cirrhotic patients is suboptimal and has a high virological relapse rate ^39– 41^. The addition of a NS5A inhibitor to this regimen could improve response rates, but the incremental efficacy of a 12-week regimen remains limited ^43^. Although the duration has not been investigated within clinical trials, current guidelines recommend a 24-week regimen including a NS5B inhibitor and a NS5A inhibitor with or without RBV. So far, none of the currently approved DAAs have optimal antiviral activity against HCV genotype 3, so the “re-introduction” of PegIFN for this genotype needs to be considered among those who are able to tolerate its side effects ^44^. Recently, a pan-genotypic regimen for 12 weeks, including a NS5B and NS5A inhibitor, seemed highly effective for HCV genotype 3, even among treatment-experienced patients with cirrhosis ^45^.

### HCV genotype 4

When PegIFN-based treatment was considered, patients with HCV genotype 4 used to be grouped with patients infected with HCV genotype 1. With the DAAs, these patients respond to the same regimens as well, and possibly even better. Although data in cirrhotic patients are scarce, due to the low prevalence of this genotype in most Western countries, the combination of a NS5B inhibitor and RBV is a plausible option. A regimen including two DAAs from separate classes (a NS5B inhibitor, a NS5A inhibitor, or a second-generation NS3/4A protease inhibitor) could also be used to eradicate chronic HCV genotype 4. When physicians want to reduce the chance of virological relapse, RBV could be added to the regimen, provided that patients are able to tolerate this.

## Decompensated cirrhosis

In general, patients with decompensated cirrhosis (Child-Pugh B/C) have lower response rates than patients with compensated cirrhosis (Child-Pugh A) ^46^. Reasons for these lower response rates may include reduced drug delivery due to shunting leading to HCV reservoirs, altered drug metabolism and uptake due to impaired liver synthetic function, or impaired immune responses which are present in cirrhotic patients ^47^.

Obviously, among patients with decompensated cirrhosis, the IFN-free regimens are far better tolerated as compared to the PegIFN and RBV combination therapy, which has been the standard of care for the last 15 years. However, as more real-world data are emerging, safety issues regarding the use of DAAs among those patients with the most advanced liver disease have arisen. Two patients with hepatic decompensation developed severe drug-induced liver injury leading to death and liver transplantation. Both patients were treated with sofosbuvir, a NS5A inhibitor, and RBV ^48^. A recent study by Welker *et al.* described the occurrence of lactate acidosis among patients treated with sofosbuvir-based regimens, with or without the addition of RBV ^49^. Whether the clinical deterioration could be attributed to the use of DAAs or RBV or whether this is merely in line with the poor natural history of patients with decompensated cirrhosis remains a matter of debate. Likewise, the occurrence of hepatic decompensation during antiviral treatment has been reported for several treatment regimens, leading the FDA to discourage the use of dasabuvir, ombitasvir, and paritaprevir/ritonavir for patients with decompensated liver disease ^50^. Also, because of the real-world safety issues which were encountered with the first-generation protease inhibitors telaprevir and boceprevir among patients with cirrhosis and low platelets or low albumin levels, one could argue that protease inhibitors may not represent an ideal class of DAAs for those with the most severe cirrhosis ^18^. Simeprevir, a first-generation second-wave protease inhibitor, has actually never been registered for patients with decompensated cirrhosis. It is clear that further studies with a focus on the safety profile of the IFN-free regimens among patients with decompensated liver disease are urgently needed. It would be highly relevant to be able to predict which of these patients can and cannot be safely treated with the IFN-free regimens.

## Resistance-associated variants

The high efficacy of IFN-free regimens will lead to high rates of SVR, even among the population that was difficult to treat and/or difficult to cure in the era of IFN-based therapy. However, as pointed out earlier, lower response rates were observed among patients with advanced liver disease. The emergence of RAVs seems a relevant factor in case antiviral therapy is not successful. Although heterogeneous methods were used to detect RAVs, it has been estimated that 53–91% of patients with virological relapse harbor HCV isolates that are resistant to one, two, or three DAAs ^51^. The presence of RAVs before IFN-free treatment initiation could be an important cause of virological failure as well, and fuels the ongoing debate of whether we should perform pretreatment viral sequencing. Relevant in this respect is that the second-generation DAAs, which are coming shortly, are thought to have a higher genetic barrier to resistance. Another option to overcome the RAVs are the advanced cellular drugs that target the host factors involved in the HCV life cycle, which have the general advantage of being pan-genotypic. Silencing of miR-122 *in vitro* showed remarkable inhibition of HCV replication and led to the possibility of targeting miR-122 as an antiviral strategy ^28, 52^. Other possible options include the HCV entry inhibitors erlotinib and dasatinib, or the cyclophilin inhibitor alisporivir ^27, 53^. In combination with DAAs, the drugs targeting host factors could be effective, especially for those patients with resistant viral strains. Still, in order to globally eradicate HCV, an effective vaccine seems necessary ^54^.

## Clinical relevance of successful antiviral therapy

In parallel with the impressive development of highly potent and well-tolerated DAAs, various cohort studies increased our understanding of the clinical relevance of these new drugs. Over the last couple of years, many researchers have published results that indicate that patients who attain SVR have a beneficial clinical outcome in terms of both liver-related and liver-unrelated endpoints. The growing body of evidence in favor of SVR is, obviously, relevant for patients and physicians. However, it is also very much needed for policy-makers who need to decide on the reimbursement of the highly effective but costly IFN-free regimens.

## Liver histology

In contrast to what was believed during the largest part of the last century, it is now widely accepted that hepatic fibrosis can regress in cases where the underlying cause of liver damage is adequately treated. Chronic HCV infection is probably the liver disease in which this is best documented. The largest histological study in which patients underwent a second liver biopsy 24 weeks after cessation of antiviral therapy indicated that, on average, the degree of hepatic fibrosis regressed among those with SVR and was rather stable among those without SVR ^55^. The most impressive result of this study, however, was that 75 of the 153 patients with cirrhosis before therapy no longer scored a METAVIR F4 in their post-treatment liver biopsy. Yet, from a previous study from Japan, we already learned that regression of hepatic fibrosis is likely to take more time ^56^. Shiratori *et al.* included 593 patients in whom the time to the post-treatment liver biopsy ranged from 1 to 10 years. Among those with SVR, the authors found that regression of fibrosis was more pronounced in cases where the biopsy was repeated after more than 3 years of follow-up. Still, even in cases with much longer follow-up, histological studies have been unable to show that all HCV-infected patients with cirrhosis who attained SVR improve their METAVIR F4 score. The concept of a point of no return with respect to the extent of liver damage seems plausible, especially because the vascular abnormalities within a cirrhotic liver have never been shown to improve, and shunting of blood through vascularized portacaval septa can lead to hypoperfusion of liver parenchyma, with hypoxemia as a contributing factor for hepatic inflammation and fibrosis ^57– 59^. On the other hand, the semi-quantitative fibrosis scores may also be somewhat too crude to appreciate all histological improvements following the eradication of HCV infection. Indeed, a recent study assessed the change in the total area of fibrosis among 38 Italian patients with cirrhosis who attained SVR ^60^. Among the minority (39%), in whom the METAVIR F4 score was not reduced after a median duration of 5.6 years in between both liver biopsies, the total area of hepatic fibrosis was still significantly reduced. While the discussion on whether cirrhosis is reversible is ongoing, it may actually be more relevant to consider the relationship between HCV eradication and the clinical sequelae of cirrhosis ^61^.

## Liver-related morbidity and mortality

Most of the Western studies that assessed the association between HCV eradication and hepatic decompensation or HCC have solely included patients with advanced liver disease, who are most at risk for these cirrhosis-related complications. However, as we have long depended on IFN-based treatment regimens, these studies predominantly included cirrhotic patients with relatively favorable characteristics. Veldt *et al.* were one of the first groups to show that the incidence in liver failure was markedly reduced among patients with chronic HCV infection and advanced liver fibrosis who attained SVR ^62^. Interestingly, this beneficial outcome was apparent immediately upon HCV clearance. Hereafter, larger studies with longer follow-up duration not only confirmed these results but also showed a significant association between SVR and a reduced occurrence of HCC, with strong hazard ratios (HRs) adjusted for many potential confounders ^63– 65^. In a recent meta-analysis, in which the results of all available cohort studies among patients with advanced liver disease were pooled, the results indicated that within this population the HR of SVR for the occurrence of HCC was 0.23 (95% confidence interval [CI] 0.16–0.35) ^66^. Combining studies that included patients with all stages of fibrosis resulted in a pooled HR of 0.24 (95% CI 0.18–0.31) regarding SVR and HCC occurrence, although these studies were mostly performed in Japan, where the incidence of HCC is substantially higher. Considering the potential benefits of SVR on these cirrhosis-related morbidities, it is not surprising that patients with cirrhosis who clear their chronic HCV infection have a reduced liver-related mortality ^65, 67^. Still, it is noteworthy that patients with advanced liver disease are not free from cirrhosis-related complications following HCV eradication. A combined cohort including 1000 patients with advanced liver fibrosis and IFN-induced SVR showed that the annual risk of HCC remains at about 1% following HCV eradication in patients with cirrhosis ^68^. The risk of HCC depends on age, the presence of diabetes mellitus, and laboratory markers of liver disease severity. One may thus expect a rising incidence of HCC following successful antiviral therapy in the era of DAAs, which will enable older patients with more advanced liver disease to attain SVR.

## Extrahepatic consequences

While a large number of studies indicated potential liver-related benefits of SVR, more recent efforts have focused on the association between antiviral therapy and extrahepatic disease. With respect to patient-reported outcomes, eradication of HCV infection decreases both the frequency and the severity of fatigue ^8^. This may be an important reason for the improved HRQoL which has been observed upon SVR ^69^. Although difficult to quantify in daily practice, these effects are probably more directly noticeable for patients and their health-care providers than the prevention of future clinical complications.

Even a few years ago, it was reported that the risk of diabetes mellitus is about three times lower among patients with SVR as compared to patients without SVR ^70^. The deteriorating consequences of diabetes mellitus are diverse but surely include renal failure and cardiovascular events. A reduced incidence of both of these solid endpoints in cases of antiviral therapy use was recently shown in a nationwide cohort study from Taiwan, in which 12,384 treated patients and 24,768 propensity score-matched untreated controls were included. The cumulative 8-year incidences of end-stage renal disease (0.15% vs. 1.32%), acute coronary syndrome (2.21% vs. 2.96%), and ischemic stroke (1.31% vs. 1.76%) were significantly lower among treated as compared to untreated patients (p<0.05 for all), and the effect of antiviral therapy remained statistically significant in multivariate analysis ^71^. These findings were immediately confirmed, and assigned to SVR, in a large population-based study which is expected to include about 80% of all IFN-treated chronic HCV-infected patients in Scotland ^72^. Apart from diabetes mellitus, we were recently presented with another possible explanation for these findings, as Gragnani *et al.* showed that HCV eradication led to the disappearance of cryoglobulinemia and resolved manifestations of the mixed cryoglobulinemia syndrome in nearly all patients ^73^. Other potential extrahepatic benefits of SVR that have been presented within recent years include a reduced occurrence of malignant lymphomas and reduced hospitalization because of acute alcohol intoxication or violence-related injuries ^72, 74^. Through these effects on extrahepatic morbidity, patients with SVR have not only a reduced liver-related mortality but also a reduced liver-unrelated mortality ^72, 75^.

## All-cause mortality

The most important new insights with regard to the clinical benefit of antiviral therapy probably concern the association between SVR and a prolonged overall survival as the most definitive clinical endpoint. In 2011, Backus *et al.* were the first to show that SVR was statistically significantly associated with a reduced all-cause mortality (HR 0.70, 0.64, and 0.51 for HCV genotype 1, 2, and 3, respectively) in a large cohort of 16,864 patients with chronic HCV infection who were followed for a median duration of 3.8 years ^76^. However, these results were derived from the specific patient population of American veterans, among which there is substantial comorbidity, risk behavior, and a rather high mortality rate. Another report extended this finding to the general HCV-infected patient population with advanced liver disease ^67^. Among the 530 patients with chronic HCV infection and advanced liver fibrosis who were followed for a median of 8.4 years, the cumulative 10-year overall survival was 91% among those with SVR, versus 74% among those without SVR (HR 0.26, 95% CI 0.14–1.49). In contrast to those who were unsuccessfully treated, patients with SVR had a survival rate that was comparable to that of the age- and sex-matched general population ^77^. Importantly, in both reports, the HR of SVR for all-cause mortality was extensively adjusted for baseline characteristics known to influence both the chance of successful IFN therapy and the long-term clinical outcome. Various representable cohorts have confirmed the strong association between SVR and reduced all-cause mortality hereafter ^72, 78^. The pooled HR of SVR was 0.50 (95% CI 0.37–0.76) based on all studies which did not specifically include patients with advanced fibrosis and 0.26 (95% CI 0.18–0.37) when only studies among those with advanced liver disease were considered ^79^.

## Decompensated cirrhosis

The above-described studies, which suggest a clinical benefit of SVR, were performed among patients treated with IFN-based regimens, so that even those included with cirrhosis had relatively favorable baseline characteristics. Indeed, in the IFN era, patients with decompensated cirrhosis were frequently withheld from antiviral therapy but were also unlikely to attain SVR if treatment was initiated. Consequently, the clinical relevance of attaining SVR is largely unknown apart from the fact that achievement of SVR would be desirable to prevent HCV recurrence in cases of liver transplantation. Because of the beneficial safety profile of the DAAs, our experience with antiviral therapy in patients with chronic HCV infection and decompensated cirrhosis is increasing rapidly. Still, because these therapeutic options have just surfaced, studies with sufficient follow-up to assess the true clinical impact of IFN-free therapy among the patients with the most advanced liver disease have to be awaited. In the meantime, several interesting observations in the short term have been presented, which focus on the Model for End-Stage Liver Disease (MELD) score. Deterding *et al.* treated 34 HCV-infected patients with Child-Pugh B/C with various IFN-free regimens. At 12 weeks post-treatment, the MELD score improved in 68%, remained stable in 23%, and worsened in 10% of patients ^80^. Based on the first experiences in England, Foster *et al.* reported the change in MELD score 4 weeks after the cessation of DAA therapy ^81^. Of the 220 patients, 105 (47.7%) had no significant change in MELD score; in 92 (41.8%) patients, MELD score improved by ≥2 points; and in 23 (10.5%) patients, MELD score worsened by ≥2 points. Additional analyses indicated that the MELD score more frequently improved rather than declined among younger patients (<65 years) and patients with a high albumin level (>35 g/L). Still, MELD improvements are mostly moderate, so the important question of whether liver transplantation can really be averted remains. If not, the slight improvement in MELD score may actually negatively impact the patient’s chances on the waiting list. Because clinical trials showed excellent SVR rates with IFN-free therapy among liver transplant recipients with chronic HCV infection, it may be questioned whether patients with decompensated cirrhosis should be treated before or after transplantation ^82, 83^. Within the next 2 years, more data will hopefully become available, which may be able to guide this decision for the individual patient. Preferably, patients with decompensated cirrhosis should therefore be treated within registry studies during the upcoming years.

## Conclusion

The implementation of IFN-free treatment regimens has broadened the horizon for patients with chronic HCV infection tremendously. Within a timeframe of 5 years, important treatment developments resulted in near-perfect SVR rates, even among patients with the most advanced liver disease. As successful antiviral therapy may be lifesaving, these developments were long awaited. Some hurdles have to be taken, however, before the health burden of this chronic disease can truly be reduced. For instance, the access to DAAs needs to be broadened so that patients can be treated regardless of the severity of hepatic fibrosis. Reducing the costs of these drugs probably remains a key factor before this goal can be achieved. As developments are still ongoing, prices will hopefully fall as a result of mutual competition. Also, it is important to increase the number of patients who are diagnosed, as the majority of patients are currently unaware of their chronic viral hepatitis. Pan-genotypic regimens are currently being evaluated in phase III trials as well, which will hopefully simplify antiviral therapy even more. Until that time, treatment selection is required and prioritizing treatment is needed to limit the economic burden. Treating those with advanced hepatic disease first seems reasonable, but remains far from ideal.

## Abbreviations

 DAAs, direct-acting antivirals; FDA, US Food and Drug Administration; HCC, hepatocellular carcinoma; HCV, hepatitis C virus; HR, hazard ratio; HRQoL, health-related quality of life; IFN, interferon; MELD, Model for End-Stage Liver Disease; miR-122, microRNA-122; PegIFN, pegylated interferon alpha; RAVs, resistance-associated variants; RBV, ribavirin; SVR, sustained virological response.
